# Conclusion of Unpublished Investigations

**Published:** 1869-02-01

**Authors:** 


### *Conclusion of Unpublished Investigations.*

BY DR. RABUTEAU.

1st. The *chlorate of potassa* is totally eliminated in its natural state. I believe that I am the first who has demonstrated this fact experimentally, by absolving chlorate of potassa and making a *quantitutive analysis* of the salt recovered in the urine. Möhler, as early as 1824, and subse-

(*) Husemann, *Handbuch der Toxicologie.*

quently other experimenters, amongst whom I would instance Justin and Isambert, had already recognized the passage of the chlorate into the urine. But doubts exist upon this subject. Some assert still, as at the beginning of the century, the metamorphosis of the salt into chloride; others, and I was of this number, believed that only a portion was reduced in the organism.

2d. *Chloric acid* in very weak doses is eliminated in the state of chloride.

3d. *Perchlorate of Potassa* is eliminated totally in its natural state. Its elimination is as rapid as that of the chlorate.

4th. The formiates and the succinates are transformed in the organisim into carbonates. It is possible that the succinates are transformed at first into malates, then into tartrates, and these finally into carbonates.

5th. Caprylic alcohol, introduced into the digestive tube, is recovered in the urine unchanged, or perhaps in the state of ether. I believe that a comparative investigation of all the alcohols, with reference to their mode of elimination most easy to determine, would throw much light upon the mode of elimination of ethylic alcohol and ordinary alcohol. *Sulphate of soda* injected into the veins in the dose of from seven to fourteen grammes, constipates and arrests thirst. The elimination by the urine, after the injection of fourteen grammes of sulphate of soda continued during two days and a half.

7th. *The hypo-sulphates of soda and magnesia* injected into the blood or ingested into the digestive tube, are eliminated principally unchanged, introduced into the blood they constipate; absorbed by the digestive tubes they act as mild purgatives.

8th. *The sulphites* are transformed into sulphates from the moment when they penetrate into the organism. Polli has already observed this metamorphosis; according to him, sulphites are found in the urine the first day, but on the second day sulphates are recognized. I determined that the salt is *eliminated totally in the form of sulphate* if the dose has been small.

8th. The *hypo-sulphites* are transformed into sulphates, and their metamorphosis commences from the moment when they have penetrated into the organism. Kletzinsky has already observed the transformation of the hypo-sulphite of soda into the sulphate.

